# Preparation and Characterization of Microcellulose and Nanocellulose Fibers from *Artemisia Vulgaris* Bast

**DOI:** 10.3390/polym11050907

**Published:** 2019-05-19

**Authors:** Kai Nie, Yan Song, Shaoyang Liu, Guangting Han, Haoxi Ben, Arthur J. Ragauskas, Wei Jiang

**Affiliations:** 1 State Key Laboratory of Bio-Fibers and Eco-Textiles, Qingdao University, Qingdao 266071, China; 15021329163@163.com (K.N.); 18765915091@163.com (Y.S.); kychgt@qdu.edu.cn (G.H.); benhaoxi@163.com (H.B.); aragausk@utk.edu (A.J.R.); 2 College of Textile & Clothing, Qingdao University, Qingdao 266071, China; 3 Department of Chemistry and Physics, Troy University, Troy, AL 36082, USA; lius@troy.edu; 4 Department of Forestry, Wildlife, and Fisheries, Department of Chemical and Biomolecular Engineering, University of Tennessee, Knoxville, TN 37996, USA; 5 Oak Ridge National Lab, Oak Ridge, TN 37831, USA

**Keywords:** *Artemisia vulgaris*, microcellulose fiber, nanocellulose fibers, natural fibers

## Abstract

*Artemisia vulgaris* is an economic plant that is spreading widely in central China. Its unused bast generates a large amount of biomass waste annually. Utilizing the fibers in *Artemisia vulgaris* bast may provide a new solution to this problem. This research attempts to strengthen the understanding of *Artemisia vulgaris* by analyzing its fiber compositions and preparing micro- and nano-cellulose fibers, which can be used as raw materials for composites. In this work, *Artemisia vulgaris* bast powder (AP) and microcellulose and nanocellulose fibers (AMFs and ANFs) were produced and characterized by optical microscopy, transmission electron microscopy (TEM), X-ray diffraction (XRD), thermogravimetric analysis (TG), and bacteriostatic test. The results indicated that cellulose, hemicellulose, and lignin were the main components in the *Artemisia vulgaris* bast. The cellulose content reached 40.9%. The *Artemisia vulgaris* single fibers were microcellulose fibers with an average length of 850.6 μm and a diameter of 14.4 μm. Moreover, the AMF had considerable antibacterial ability with an antibacterial ratio of 36.6%. The ANF showed a length range of 250–300 nm and a diameter of 10–20 nm, and it had a higher crystallinity (76%) and a lower thermal stability (initial degradation temperature of 183 °C) compared with raw ANF (233 °C). This study provides fundamental information on *Artemisia vulgaris* bast cellulose for its subsequent utilization.

## 1. Introduction

Natural fibers are composed of cellulose, which is the most abundant renewable biomaterial on earth and is completely biodegradable [1,2]. *Artemisia vulgaris* likely originated from eastern Asia [3] and is a very common weed with a strong aroma. Most *Artemisia* species are found growing abundantly throughout temperate climate areas and are cold-resistant [4]. They are widely distributed in most provinces and autonomous regions in China. Their extracts have been used throughout history to control insect pests [5]. At present, *Artemisia vulgaris* is mainly used in health care products and function foods because of the benefits of its volatile oils and flavonoids. There have been many reports on *Artemisia vulgaris*’ medicinal properties, chemical compositions, pharmacological effects, clinical applications, functional food applications, as well as processes and quality control methods [6]. However, all of the above applications only used its leaves, and the other parts were abandoned as wastes.

It should be noticed that *Artemisia vulgaris* bast contains a considerable amount of cellulose, which could be a good source of microcellulose and nanocellulose fibers. Microcellulose fibers from agricultural residues can be used as a reinforcing material in polymer composites, which have attracted increasing interest in recent years [7,8,9,10]. Microcellulose fibers can also be used in biomedicine, paper packaging, food additives, electronic devices, and many other fields [11]. Nanocellulose fiber is a renewable nanomaterial with superior properties, such as unique optical, rheological, and mechanical properties. It is light, biodegradable, biocompatible, and recyclable, which provide it with a bright future for a wide range of applications [12,13,14].

However, there is little research on the utilization of *Artemisia vulgaris* residues, especially the abundant cellulose in its bast. The production and characterization of mirco- and nanocellulose fibers from *Artemisia vulgaris* may help utilize its waste bast and improve its economic feasibility. In addition, the cellulose fibers separated from *Artemisia vulgaris* may still retain some functions of its leaves, which might add extra value to the fibers. 

In the current work, micro- and nanocellulose fibers were produced from *Artemisia vulgaris* bast. The fiber properties, including their antibacterial function, were characterized. This study provides a deeper understanding of *Artemisia vulgaris* cellulose fibers and their potential applications.

## 2. Materials and Methods 

### 2.1. Materials

*Artemisia vulgaris* bast were purchased from Linyi City, Shandong Province, China. The samples were cut into 1–2 cm lengths and dried at 105 °C before the experiment. Laboratory degrade reagents (including benzene, ethanol, ammonium oxalate, sodium hydroxide, concentrated sulfuric acid, sodium chlorite, and glacial acetic acid) were used in this work.

### 2.2. Methods

**Figure 1:**
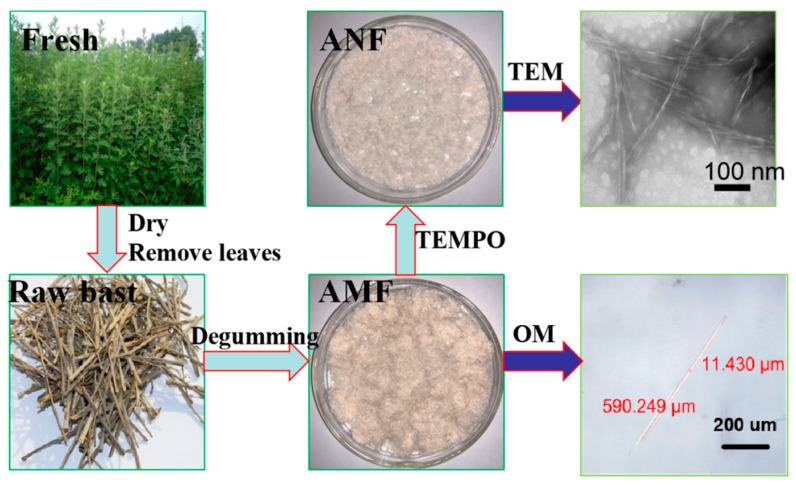
Flowchart of the *Artemisia vulgaris* bast microcellulose fiber (AMF) and *Artemisia vulgaris* bast nanocellulose fiber (ANF) extraction. (TEM, transmission electron microscopy. OM, optical microscopy. TEMPO, 2,2, 6,6-tetramethylpiperidine-1-oxyl).

Figure 1. briefly introduces the experimental route of this research. The fresh *Artemisia vulgaris* plant straw is dried and the bast is taken out. The micron-sized fiber (AMF) is prepared by degumming with alkali, and the nano-sized fiber (ANF) is prepared by oxidation of TEMPO. In order to understand the size information of products, TEM and OM characterization tests were performed on AMF and ANF.

#### 2.2.1. Determination of Chemical Composition of the Bast

According to China’s National Food Safety Standard (GB5009.3—85), a gravimetric method was used to determine the moisture content of the *Artemisia vulgaris* bast samples. The samples were processed by following China’s National Textile Standard (GB5889—86) to determine major chemical compositions, including lipid, water-soluble substances, pectin, lignin, hemicellulose, and cellulose, of the *Artemisia vulgaris* bast. All experiments were performed independently in triplicate and the results given are the means of the results.

#### 2.2.2. Preparation of *Artemisia Vulgaris* Bast Cellulose Fiber (Microcellulose Fiber)

In order to obtain *Artemisia vulgaris* bast cellulose powder (AMF), the dried bast was cut, crushed, and passed through a 60 mesh screen. In the experiment, 2.0 g of *Artemisia vulgaris* bast powder was extracted for 3 h with a mixture of 40 mL benzene and 20 mL ethanol at 90 °C in a Soxhlet extractor. The products were washed with methanol and deionized water. A bleaching process was conducted with 0.24 g of sodium chlorite and 0.2 mL of glacial acetic acid in 30 mL aqueous solution under aeration for 3 h at 75 °C (adding the same amounts of chemicals per hour). The bleached bast powder was boiled in an NaOH aqueous solution (20 g/L) for 3 h, then washed until neutral to obtain *Artemisia vulgaris* bast cellulose powder (AMF) [15].

#### 2.2.3. Preparation of *Artemisia Vulgaris* Bast Nanocellulose Fiber

First, 1.0 g of the *Artemisia vulgaris* bast cellulose powder (AMF) was soaked in 30 mL dimethyl sulfoxide (DMSO, which has a certain swelling effect on cellulose) for 5 h while stirring at 600 r/min at 60 °C, filtered, and washed 3 times with distilled water, and then dried to a constant weight. After, the treated powder was added to a mixture of TEMPO (2,2, 6,6-tetramethylpiperidine-1-oxyl) (0.03%) and KBr (0.6%) at a bath ratio of 1:100 and placed at 4 °C for 12 h. Next, sodium hypochlorite (0.4%) was added into the bath while stirring for 2 h. The pH was adjusted to 10.5 with 0.1 mol/L hydrochloric acid during the whole 2 h reaction. Methanol was added to terminate the reaction when the pH stabilized. Finally, the suspension of *Artemisia vulgaris* bast cellulose powder was obtained by adjusting pH to neutral with 0.1 mol/L hydrochloric acid. The suspension was then centrifuged at 12,000 r/min for 10 min in a high-speed centrifuge. The upper supernatant was removed, fresh distilled water was added, and the centrifuging was repeated 6 times to obtain a gelatinous precipitate. The precipitate was treated in distilled water at 4 °C at a bath ratio of 1:100 and dispersed at a speed of 15,000 r/min for 5 min using a high-speed disperser to obtain an *Artemisia vulgaris* nanocellulose fiber suspension. The suspension was frozen at −20 °C for 12 h, then freeze-dried for 24 h [16].

#### 2.2.4. Bacteriostatic Test and Analysis

According to Chinese National Standard (Evaluation of antibacterial properties of textiles) (GB/T 20944), an oscillating method was used to determine the antibacterial properties of raw bast, AMF and ANF. Cotton fiber without antibacterial activity was selected as the control. The selected strain was *Escherichia coli*, and all samples and equipment were subjected to high-temperature and high-pressure sterilization before use. Two parallel samples were tested of each type of material. The formula for calculating the bacteriostatic rate is

(1) $$Br\%=\left(\frac{Qc-Qx}{Qc}\right)$$

where *B*r% is bacteriostatic rate; *Qc* is the colony quantities of cotton fibers; and *Qx* is the colony quantities of samples (raw bast, AMF, ANF).

#### 2.2.5. Optical Microscopy (OM) Analysis

The appearance and fiber dimensional size of the AP (*Artemisia vulgaris* bast powder), AMF, and ANF samples were observed by optical microscopy (OM, LeicaDM2700M, Wetzlar, Germany). 

#### 2.2.6. SEM Analysis

The microstructure, surface morphology, and cross-section of the nanocrystal cellulose were observed by scanning electron microscopy. After being coated with gold, samples were observed and photographed with the microscope operating at 10 kV (SEM, JEM-1200EX, Tokyo, Japan). 

#### 2.2.7. TEM Analysis

*Artemisia vulgaris* bast nanocellulose fiber samples were monitored by transmission electron microscopy (TEM, JEM-1200EX microscope, Tokyo, Japan) to measure its size. One percent of the ANF liquid was dispersed with ultrasonic equipment. One drop of the suspension was placed on carbon film-covered copper grids (400 mesh). The grid was dried in an oven at 70 °C before being observed and photographed.

#### 2.2.8. FTIR Analysis

In order to obtain the chemical composition changes of *Artemisia vulgaris* bast fibers, samples of *Artemisia vulgaris* bast, *Artemisia vulgaris* bast cellulose, and *Artemisia vulgaris* bast cellulose nanocrystal were investigated with Fourier transform infrared spectroscopy (FTIR, Thermo Fisher Scientific NICOLET 5700, Waltham, MA, USA) in a wavenumber range of 500–4000 cm^−1^. A KBr disc mixed with 1% milled samples was pressed before the FTIR analysis.

#### 2.2.9. XRD Analysis

An X-ray diffraction (XRD) test was carried out to obtain the change in crystallinity index (CrI) of cellulose samples during the preparation process. The crystallinity index of samples was calculated using the height ratios between the intensities of the crystalline peak (I002-Iamorph) and total intensity (I002) [17,18]. The samples were scanned by a Bruker D8 Advance X-ray diffractometer (under a voltage of 40 kV and a current of 50 mA) (Bruker, Karlsruhe, Germany) in the range of 5° to 50°. The maximum intensity of the natural cellulose crystallinity lattice (002) diffraction was taken at 2θ = 22°, and the maximum intensity of the amorphous phase was taken at 2θ = 18° [19].

#### 2.2.10. TG Analysis

In order to understand the change in thermodynamic properties during the preparation process of ANF, thermogravimetric analysis was carried out from room temperature to 700 °C under nitrogen atmosphere with a constant heating rate of 10 °C/min on a TGA (Pyris, PerkinElmer, Waltham, MA, USA).

## 3. Results and Discussion

### 3.1. Chemical Compositions Analysis

The major chemical composition of *Artemisia vulgaris* bast included cellulose, lignin, and hemicellulose, which composed 40.92%, 28.25%, and 29.10% of the bast (*w/w*), respectively, as shown in Table 1. Collectively, they accounted for 98.2% of the bast. In Chinese medicine producing processes, producers often discard the cellulose in *Artemisia vulgaris* after extracting pharmaceutical ingredients. This is a waste of the most abundant natural resource in the bast; the residue from the process may extract valuable cellulose with functions such as antibacterial abilities. The cellulose content of *Artemisia vulgaris* was equivalent with hardwood (43.71%), and a little lower than kenaf (56.81%), as listed in Table 1. It is known that kenaf and wood are two common materials to produce cellulose fibers to make composite [20,21,22,23]. Therefore, *Artemisia vulgaris* bast would also be a promising resource to make micro- and nanocellulose fibers. 

### 3.2. Morphology of the Samples and Powder/Fiber Size Analysis

**Figure 2:**
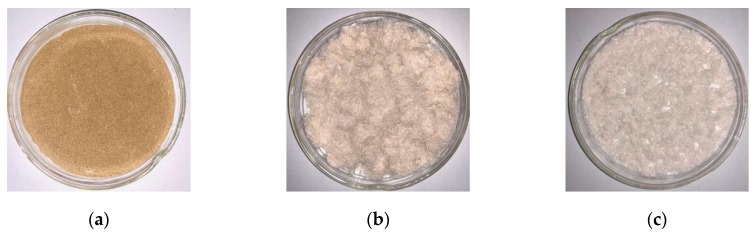
Photographs of (**a**) raw *Artemisia vulgaris* bast powder; (**b**) *Artemisia vulgaris* bast microcellulose fibers; (**c**) freeze-dried powders of nanocellulose fibers.

Figure 2 shows the appearance of freeze-dried samples of AP, AMF, and ANF. The raw *Artemisia vulgaris* bast powder was light yellow, which contained more plant phloem substances. After the bleaching and degumming processes, the color of the AMF became whiter, and rod-like substances increased; the ANF looked even finer and the size of the fiber was further reduced.

**Figure 3:**
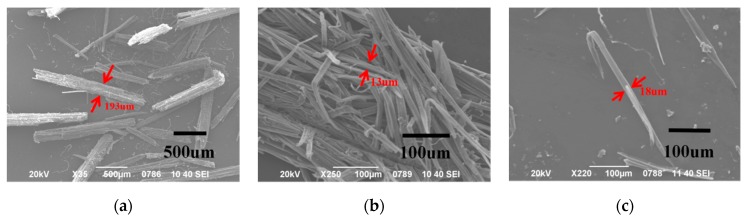
SEM images of (**a**) *Artemisia vulgaris* bast; (**b**,**c**) *Artemisia vulgaris* bast microcellulose fibers.

As illustrated in Figure 3, the surface of the raw AP was very rough, and large number of gum materials were attached to it. During the production of AMF, a large number of colloids, e.g., pectin, lignin, and hemicellulose, were removed through the treatment, and many finer fibers were obtained (Figure 3b). The NaOH boiling separated the raw fibers and shortened them. To determine the length and fineness of AMF, 100 single fibers were randomly selected and measured under an optical microscope. The dates of length, diameter, and aspect ratio of micro- and nano-fibers are shown in Table 2. Most fibers had lengths between 600–1000 μm. The averages of the length and diameter of the fibers were 850.6 ± 119.1 μm and 14.3 ± 1.9 μm, respectively. The aspect ratio was about 60. Although it is too short for the traditional textile industry, the fiber is very suitable for making nanocellulose. The cost and difficulty of micron size to nanometer size is greatly reduced with this fiber length. The larger aspect ratio could provide a large specific surface area, which could improve the reinforcing effect for polymer composite application [24].

**Figure 4:**
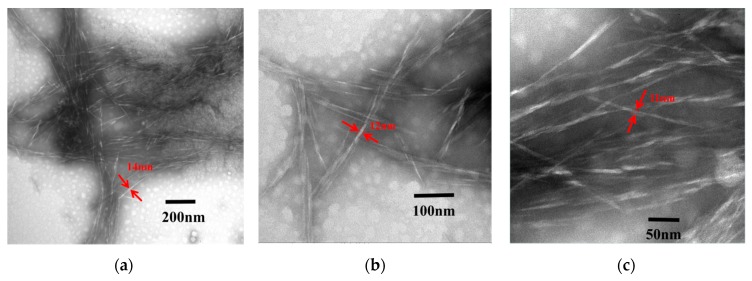
TEM images under different ruler conditions ((**a**), 200 nm; (**b**), 100 nm; (**c**), 50 nm) of *Artemisia vulgaris* bast nanocellulose fibers.

The morphology analysis of the ANFs was carried out with a TEM (Figure 4). It can be observed that the ANFs obtained through TEMPO (2,2, 6,6-tetramethylpiperidine-1-oxyl) oxidative hydrolysis were rod-shaped and evenly dispersed. To analyze the size of ANFs, 100 single fibers were randomly selected and observed. The typical size of an individual ANF was about 250–300 nm long and 10–20 nm wide with an aspect ratio of 15–25. 

### 3.3. Antibacterial Properties

**Figure 5:**
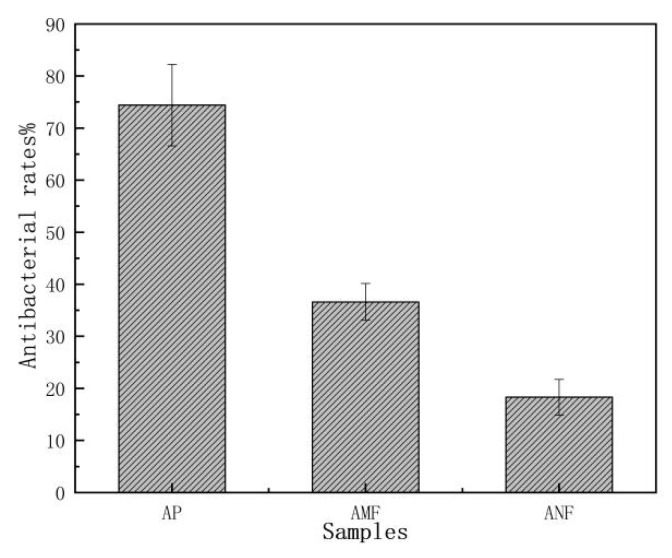
The antibacterial rates of *Artemisia vulgaris* bast powder, microcellulose fibers, and *Artemisia vulgaris* bast nanocellulose fibers.

*Artemisia vulgaris* is well known by its high antibacterial property, but the data were mostly obtained from the extracts of its leaves [25]. The study of the antibacterial ability [26,27] of raw bast powder, AMF and ANF, would offer additional information for their potential functional applications. Figure 5 shows the bacteriostatic rates of the three samples. The original *Artemisia vulgaris* bast powder had good antibacterial activity against *Escherichia coli*, and the antibacterial rate reached 76%. After the degumming and TEMPO oxidation processes, the antibacterial rates of AMF and ANF decreased to 36.6% and 18.3%, respectively. It suggests that the effective ingredients against bacteria were partially removed by the treatments. However, some active ingredients did remain on the fibers and they still maintained an extent of antibacterial ability. So, the AMF and ANF from *Artemisia vulgaris* would be especially useful for applications preferring antibacterial function, which may add extra value to the fibers.

### 3.4. FTIR Spectroscopy Analysis

**Figure 6:**
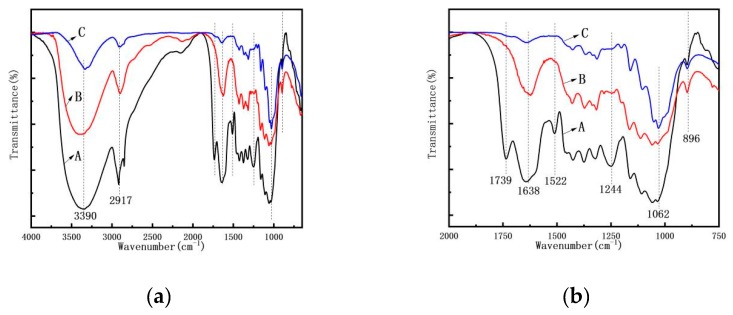
The FTIR spectra (**a**) of (A) *Artemisia vulgaris* bast powder; (B) *Artemisia vulgaris* bast microcellulose fiber, and (C) *Artemisia vulgaris* bast nanocellulose fiber. (**b**) A clearer view of peak changes in the 750–2000 cm^−1^ range.

To understand the chemical composition change during the process, FTIR spectra of the AP, AMF, and ANF were obtained (Figure 6). The composition change was manifested by the change of certain absorption peaks which can be linked to certain components in the samples [28,29]. The absorption peak at 1062 cm^−1^ represents the C–O single bond in cellulose [30]. Their relative intensities did not change after the treatments, suggesting the cellulose in the *Artemisia vulgaris* did not change or degrade during the process. On the other side, the absorption peak at 1739 cm^−1^, which corresponds to the C=O bond in pectin or hemicellulose, disappeared on the AMF and ANF spectra. This indicates that the pectin and hemicellulose contents were effectively removed by the treatments [31]. The peaks at 1244 cm^−1^, 1522 cm^−1^, and 1638 cm^−1^ represent the C–O stretching vibration, benzene ring vibration, and C=C stretching vibration, respectively, in lignin. They significantly were reduced during the process, suggesting a large portion of lignin was removed. The β-(1, 4) glycosidic linkage between the monosaccharides of the cellulose characteristically peaked at 896 cm^−1^. In summary, cellulose was retained during the process while other components in the bast, e.g., hemicellulose and lignin, were effectively removed by the treatments.

### 3.5. Crystallinity Index of Samples

**Figure 7:**
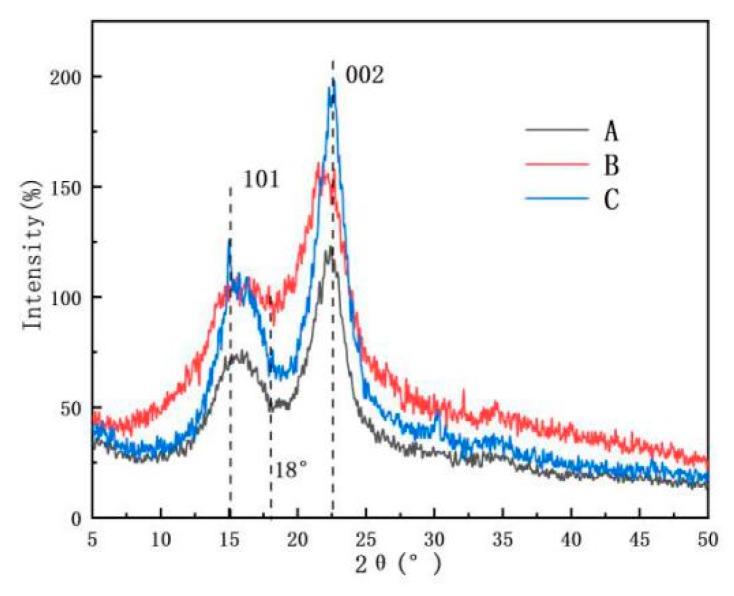
XRD spectra of (A) *Artemisia vulgaris* bast powder; (B) *Artemisia vulgaris* bast microcellulose fiber, and (C) *Artemisia vulgaris* bast nanocellulose fiber.

The XRD spectra of AP, AMF, and ANF are shown in Figure 7. All the samples had diffraction peaks of 2θ at 15.1° and 22.5°, which were assigned to the (110) and (200) planes of cellulose crystal, respectively [32,33,34]. It confirmed that the cellulose *I* crystal structure of cellulose did not degrade during the process. The crystallinity index related to mechanical properties were calculated to be 36.72%, 66.71%, and 76.33% for AP, AMF and ANF, respectively. The higher crystallinity of the nanocellulose was because of the removal of amorphous components, such as hemicellulose, lignin, and less-perfect regions of cellulose.

### 3.6. TG Analysis

**Figure 8:**
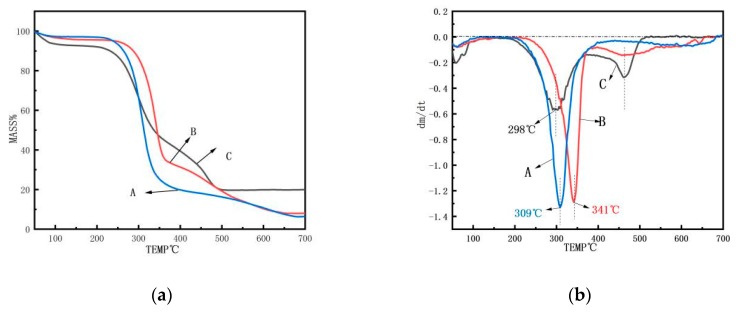
(**a**) TG spectra and (**b**) the first derivative TG thermograms of (A) *Artemisia vulgaris* bast powder; (B) *Artemisia vulgaris* bast microcellulose fiber, and (C) *Artemisia vulgaris* bast nanocellulose fiber.

The thermal stability of AP, AMF, and ANF under a nitrogen environment was investigated by thermogravimetric (TG) analysis, as illustrated in Figure 8. The analysis of the thermal stability of the fibers was essential to characterize the limit of their processing temperature for various applications [35]. The initial degradation temperature, the maximum degradation rate temperature, and the termination degradation temperature of the ANF were 183 °C, 298 °C, and 485 °C, respectively, which were far lower than those of the AMF (233 °C, 341 °C, and 655 °C, respectively). Regarding the mass change rate (Figure 8b), the degradation rate of ANF was much lower than those of AP and AMF. Figure 8a also shows that ANF had about 21% mass residue after the thermal degradation, which was much higher than the other two samples. The slower degradation rate (between about 200–500 °C) and higher residue were probably due to the removal of amorphous regions in the ANF cellulose, which only left well organized crystal areas in the fibers.

The thermal stability change was probably due to the presence of three main reasons: First, compared to AMF, the particle size of ANF was reduced, the specific surface area was increased, and the exposed carbon and reactive group activity were increased. Second, the fiber segment was broken by the treatments, which caused low molecular weight segments and breakpoint defects in ANF [36,37]. They were more susceptible to thermal degradation. Last, the lower stability of TEMPO-oxidized cellulose nanofibers might also be attributed to the introduction of the carboxylate group on the cellulose surface [38]. As a result, the thermal stability of ANF was lowered.

## 4. Conclusions

In this work, the micro- and nanocellulose fibers of *Artemisia vulgaris* bast were successfully obtained through an alkali degumming process and TEMPO chemical treatments, respectively. The FTIR and XRD analyses showed that non-cellulosic components were effectively removed during the process and the crystallinities of the samples were gradually increased. It was found that the obtained microcellulose fiber had an average length and diameter of 850 μm and 14.3 μm, respectively, while the nanocellulose fibers showed a length range of 250–300 nm and a diameter of 10–20 nm with good thermal property and high crystallinity. Both micro- and nano-fibers retained certain antibacterial properties. The results suggest that *Artemisia vulgaris* bast waste is a good natural resource for efficient production of micro- and nanocellulose fiber with good performance, which could provide additional value to the economic plant.

## Figures and Tables

**Table 1:** Chemical compositions of *Artemisia vulgaris* bast, kenaf bast, and hardwood.

Species	Hemicellulose	Lignin	Cellulose	Lipid	Water Soluble Matter	Pectin	Gum Content
*Artemisia vulgaris* bast	29.10% ± 0.97%	28.25% ± 0.45%	40.92% ± 0.70%	1.05% ± 0.04%	13.6% ± 2.41%	3.2% ± 0.34%	35.90% ± 1.16%
Kenaf bast [20,21,22]	15.75% ± 1.07%	15.65% ± 1.53%	56.81% ± 2.95%	8.42% ± 0.08%	6.28% ± 0.23%	4.43% ± 0.39%	28.46% ± 0.95%
Wood [23]	35.97% ± 3.88%	26.24% ± 2.41%	43.71% ± 2.10%	2.11% ± 1.42%	-	-	-

**Table 2:** The length and diameter of micro- and nano-fibers.

Species	Length				Diameter				Aspect Ratio
	Avg.	Max.	Min.	SD	Avg.	Max.	Min.	SD	
AMF	850.6 μm	1212 μm	400.2 μm	119.1	14.3 μm	15.8 μm	12.3 μm	1.9	59.5
ANF	260.5 nm	307.7 nm	207.7 nm	33.6	11.4 nm	15.7 nm	7.7 nm	1.9	22.8
